# A comparison of non-fatal self-poisoning among males and females, in Sri Lanka

**DOI:** 10.1186/s12888-014-0221-z

**Published:** 2014-08-08

**Authors:** Thilini Rajapakse, Kathleen Margaret Griffiths, Helen Christensen, Sue Cotton

**Affiliations:** Department of Psychiatry, Faculty of Medicine, University of Peradeniya, Peradeniya, Sri Lanka; National Institute for Mental Health Research, The Australian National University, Building 63, Canberra, ACT 0200 Australia; Black Dog Institute, University of New South Wales, Hospital Road, Randwich, NSW 2013 Australia; Centre for Youth Mental Health, University of Melbourne, 35, Poplar Road, Parkville, VIC 3052 Australia

## Abstract

**Background:**

In the recent past Sri Lanka has had a high rate of attempted suicide by pesticide ingestion, among both males and females. Recent evidence suggests that these trends in self-poisoning may be changing, with increasing medicinal overdoses and changing gender ratios. In the past, attempted suicide in Sri Lanka has been described as impulsive acts, but research regarding aspects such as suicidal intent is limited, and there has been no comparison between genders. The objective of this study was to describe gender differences in non-fatal self-poisoning in Sri Lanka with respect to substances ingested, triggers, stressors, suicidal intent and psychiatric morbidity.

**Methods:**

Persons admitted to Teaching Hospital Peradeniya, Sri Lanka, for medical management of non-fatal self-poisoning over a consecutive 14-month period were eligible for the study. Participants were interviewed within one week of admission, with regard to demographic details, poison type ingested, triggers, psychiatric morbidity and suicidal intent. 949 participants were included in the study, of whom 44.2% were males, with a median age of 22 years.

**Results:**

Males were significantly more likely to ingest agrochemicals, whereas females were more likely to overdose on pharmaceutical drugs. Interpersonal conflict was a common trigger associated with non-fatal self-poisoning for both males and females. Alcohol use disorders and high suicidal intent were significantly more likely in males. There was no difference in rates of depression between the genders. Multiple regression for both genders separately showed that the presence of depression and higher levels of hopelessness was the strongest predictor of suicidal intent, for both genders.

**Conclusions:**

Patterns of non-fatal self-poisoning in Sri Lanka appear to be changing to resemble Western patterns, with females having a greater rate of self-poisoning and more medicinal overdoses than males. Alcohol use disorder is a gender specific risk factor associated with non-fatal self-poisoning among males, indicating a need for specific intervention. However there are also many common risk factors that are common to both genders, particularly associations with interpersonal conflict as an acute trigger, and psychiatric morbidity such as depression and hopelessness being related to increased suicidal intent.

## Background

Non-fatal self-poisoning is a challenging problem facing Sri Lanka, as well as other South Asian countries [1,2]. The annual cost of treating self-poisoning patients in Sri Lanka in 2004 was estimated to be as high as $866,304 [3]. Previously, the commonest method of self-poisoning in Sri Lanka was by ingestion of pesticides, and rates were equal or higher in males compared to females [4,5]. This gender ratio contrasted with international studies which report higher rates of attempted suicide by drug overdoses among females [6], but was consistent with the reports from other developing countries where the gender ratio for non-fatal self-poisoning by pesticide ingestion is roughly 50/50 [7-12]. However, this gender pattern now appears to be changing in Sri Lanka, with more women being admitted to hospital as a result of the ingestions of medicines and other substances [13,14]. Similar patterns of change have been reported in other countries in the Asian regions such as Pakistan and Vietnam [15,16]. With increasing urbanization, patterns of non-fatal self-poisoning in Sri Lanka and other South Asian countries may be changing to resemble that of developed countries, accompanied by an increasing proportion of attempted suicides among females [13-16].

Rates of non-fatal self-poisoning appear to be increasing in Sri Lanka [1], with changing patterns in recent years as described above. Despite this, there is little information about the suicidal intent, and risk factors associated with self-poisoning attempts, among men and women in Sri Lanka. In the West, greater suicide intent has been reported in men than women, and this may partly account for the greater proportion of attempts relative to completions in women [17,18]. International studies have reported age, childhood adversity, recent life events and psychiatric morbidity such as depression, hopelessness and alcohol misuse, to be associated with increased risk of suicidal attempts among men and women [19-25]. International studies also indicate that violent methods of self-harm, alcohol use disorders and economic stressors are associated with suicide attempts in males, whereas a history of sexual trauma and anxiety disorders are commonly associated with suicide attempts among females [17,26,27]. Available evidence suggests that non-fatal self-poisonings in Sri Lanka are impulsive acts [28], but these findings are limited by the lack of formal assessment of suicidal intent. There has been no comparison between genders with regard to non-fatal self-poisoning in Sri Lanka.

Thus, the overall objective of this study was to describe gender differences in non-fatal self-poisoning in Sri Lanka, especially given the changing gender patterns in recent years. We anticipate that a better understanding of the factors such as psychiatric morbidity, suicidal intent, and life stressors associated with non-fatal self-poisoning in men and women separately, would point the direction for gender specific intervention strategies, applicable for Sri Lanka and perhaps other South Asian countries as well.

For the purposes of this study, non-fatal self-poisoning was defined as an act where a person deliberately ingested a poisonous substance, or ingested a medicinal drug in excess of prescribed dosage, with a non-fatal outcome. Our first analysis involved the examination of whether there were any gender differences in the characteristics of non-fatal self-poisoning in terms of substances ingested, triggers, psychiatric morbidity and suicidal intent associated with the act. The second analysis examined factors that predicted suicidal intent in males and females.

## Methods

### Participants and setting

The study was carried out at the toxicology unit (ward 17) of Teaching Hospital, Peradeniya, Sri Lanka, from February 2012 onwards, over a consecutive 14-month period. Teaching Hospital Peradeniya is a tertiary care hospital, situated in the second largest city in the Central Province of the country. All persons who present to Teaching Hospital Peradeniya for medical management of acute self-poisoning (who do not need intensive unit (ICU) care), are admitted to the toxicology unit, and those admitted directly to the ICU are subsequently transferred after recovery to the toxicology unit. This includes persons residing in urban and semi-urban areas around Peradeniya, as well as persons transferred from hospitals in rural agricultural areas in the central and north-central areas of the country, since the toxicology unit at Peradeniya Hospital serves as a referral unit for other hospitals in these areas.

All persons admitted to this unit, due to non-fatal self-poisoning, aged 14 years and above, who were conversant in Sinhalese or/and English, were eligible for this study. Potential participants were approached by the researcher and informed about the study within one week of admission to hospital. Those who gave informed written consent were enrolled. Exclusion criteria were inability to speak either Sinhala or English, and being too medically unwell to participate in the interview while in hospital. Ethical clearance for the study was obtained from the Faculty of Medicine, University of Peradeniya, Sri Lanka, and the Australian National University.

### Tools and outcome measures

Details of the non-fatal self-poisoning act, nature of substances ingested and socio-demographic details were assessed by the use of an interviewer administered questionnaire. Certain parts of the survey, such as the section on socio-demographic details were similar to those used in the World Health Organization (WHO) Supre-Miss study [29].

Suicidal intent associated with the act was assessed based on the total score of the Pierce Suicide Intent Scale (PSIS). The PSIS is an interviewer administered standardized tool [30] which has been used previously in Sri Lanka [31]. The PSIS comprises three sections, namely: a section on *circumstances* related to the suicide attempt which includes six items regarding aspects such as planning of the act and precautions against being found out (each scored as 0,1,2); a section for *self-report* which includes four items about the person’s self-report regarding intention, understanding of lethality and premeditation; and a section on *risk*, which consists of two items regarding the medical lethality of the act [30]. The total score is obtained by summing scores from all three sections. Total scores on the PSIS range from 0 to 21, and the total scale score can also be categorized as follows: low (total score 0–3), medium (total score 4–10) and high (total score >10) suicidal intent [30].

Depression was assessed using two self-report screening tools, namely the Peradeniya Depression Scale (PDS) and the Patient Health Questionnaire (PHQ-9) [32,33]. The PDS has been created and validated for screening of depression in a Sinhala-speaking Sri Lankan population [33]. The PHQ-9 was used because it is an internationally validated screening tool, although at the time of conducting this study, the PHQ-9 had not been validated for use in Sri Lanka [32]; therefore it was translated from English into Sinhala and back-translated prior to use. A total score of ≥ 10/25 was considered positive for the screening of depression by the PDS [33], and a score of ≥ 10 was considered positive for the screening of moderate or severe depression by the PHQ-9 [32]. The Generalized Anxiety Disorder Screener (GAD-7) [34] was used to screen for anxiety. This measure was translated into Sinhala and back translated prior to use.

Alcohol misuse was assessed by using the Alcohol Use Disorders Identification Test (AUDIT), which has been validated in Sinhala for a Sri Lankan population [35,36]. The AUDIT screens for hazardous drinking (score ≥7) defined as ingestion of 60 g or more of alcohol per occasion, and for alcohol use disorder (score ≥16) which includes harmful use and alcohol dependency (according to the ICD-10 Classification system) [35].

Significant life events in the six months prior to the act of non-fatal self-poisoning was examined using a questionnaire derived from the Bughra Life Threatening Events Questionnaire [37]. The Beck Hopelessness Scale (BHS) was used to assess the degree of hopelessness [23]. Both the BHS and the Bughra Life Threatening Events Questionnaire were translated into Sinhala and back translated prior to use. In most cases these questionnaires were self-administered, and the participant was given time to complete them without the interviewer being present. However if the participant was unable to read the questionnaire, the interviewer read out each statement aloud and ticked the relevant response as directed by the participant.

### Procedure

In most cases, participants were interviewed within one week of admission to hospital. The interview was carried out in two 40-minute sessions and comprised several questionnaires administered by the researcher and several self-administered screening tools. Most interviews and assessments were conducted in the Sinhala language, except in instances when English was the participant’s preferred choice of communication.

### Statistical analysis

Data analyses were undertaken using IBM® SPSS® Statistics Version 21.0. Data screening was undertaken to check for outliers and for assumption testing (e.g., deviations from a normal Gaussian distribution and violations to heterogeneity to variance). Data screening entailed examination of descriptive statistics (e.g., skewness and kurtosis) and inspection of histograms and boxplots. Appropriate measures such as transformations or adoption of non-parametric statistics (e.g., Mann–Whitney U test) were undertaken as needed.

To determine the relationship between gender and characteristics of non-fatal self-poisoning, logistic regressions were used to determine the odds ratio (OR) and the 95% confidence interval of the OR. The significance of the associations was determined using the Wald statistic. When required, additional analyses were conducted using chi-square test (χ^2^) for categorical self-poisoning measures and independent t-tests for continuous self-report measures. Significant χ^2^ results for cross-tabulations greater than a 2 × 2 table were explored more closely with post hoc pairwise comparison of column proportions using the Bonferroni correction. For the sake of clarity in reporting, with regards to gender differences of categorical variables with more than two levels, chi-square and tests of differences in proportions have been reported in the text, while the odds ratios are given in the tables.

Hierarchical multiple regression was used to determine predictors of suicide intent (PSIS). Separate models were examined for males and females. Examination of bivariate correlations and tolerances (1-SMC) indicated that there were no problems of collinearity or multicollinearity amongst predictors. As discussed earlier, factors known to be associated with attempted suicide from previous international studies [19-25], were entered into the model in a thematic hierarchical manner. Two regression models were developed, one each for males and females. The final regression model included the following sets of independent variables, entered in a thematic hierarchical manner, in the following order: socio-demographic details (age, marital status), childhood adversity (2 items), stressors in the prior six months (4 items), prior help seeking behavior or suicidal behavior (3 items), acute triggers (1 item) and psychiatric morbidity (presence of anxiety, depression and BHS scores). From these models, it was possible to ascertain the overall amount of variability explained in suicidal intent by the model, which sets of variables added significantly to the prediction of suicidal intent as well as the relative importance of individual predictors. Given the exploratory nature of the study, alpha (α) was set at .05 for all analyses. No adjustments were made for multiple comparisons because they can result in a higher type II rate, reduced power, and increased likelihood of missing important findings [38].

## Results

### Sample characteristics

A total of 1334 persons met eligibility criteria to be included in the study, of whom 9.1% (*n* = 121) refused consent, and 19.8% (*n* = 264) could not be included because they either left hospital before the interviews could be conducted, or they were in hospital but were too physically unwell to participate. A total of 949 participants took part in the study, of which 44.2% (*n* = 419) were males. The median age was 22 years, and 61.0% (*n* = 570) of the participants were below the age of 25 years. Females were significantly younger than males (median age for females: 20 years, for males: 25 years, Mann–Whitney U *z* = −7.87, *p* < .001).

There were no significant gender differences with regard to marital status or with whom the participant resided (Table 1). Females had a higher overall level of education, but were significantly less likely to be employed compared to males, OR 0.09 (95% CI .07, .12) (Table 1). The Population and Housing Census of 2001 for Sri Lanka reports that more males complete up to grade 10, but more females complete the G.C.E. (O/L) and G.C.E. (A/L), whilst males are more likely to be employed than females in all age groups (percentage employed in amongst those aged ≥10 years: males 65.5%, females 28.1%).

**Table 1:** **Socio-demographic characteristics of the total cohort and for males and females**

**Variable**	**Total sample (n = 949)**	**Gender** ^**1**^		**p value**	**Odds ratio**	**95% CI of OR**
	**% (n)**	**Male (n = 419)**	**Female (n = 530)**			
**Living with:**				.345		
Parents	52.5% (498)	50.5% (209)	55.0% (289)	na^2^	na	na
Spouse/children	40.3% (382)	42.5% (176)	39.2% (206)	.224	0.85	0.65-1.11
Other	6.2% (59)	7.0% (29)	5.7% (30)	.293	0.75	0.44-1.28
**Married**	42.9% (406)	44.7% (187)	41.4% (219)	.303	0.87	0.67-1.13
**Highest level of education:**				<.001		
No schooling/up to grade 5	5.5% (52)	9.4% (39)	2.5% (13)	na^2^	na	na
Grade 6 to 10	50.2% (476)	58.0% (240)	44.9% (236)	.001	2.95	1.54-5.67
Completed O/L Exam	28.9% (274)	25.1% (104)	32.3% (170)	<.001	4.90	2.50-9.62
Completed A/L Exam	13.7% (130)	7.2% (30)	19.0% (100)	<.001	10.00	4.73-21.14
University/Postgraduate	0.8% (8)	0.2% (1)	1.3% (7)	<.001	21.00	2.36-187.13
**Occupational status**						
Employed	41.9% (398)	72.3% (298)	19.1% (100)	<.001	0.09	0.07-0.12

^1^Gender is coded as 0 Male 1 Female.

^2^Not applicable as reference category.

### Substances used for non-fatal self-poisoning

The details of substances used for non-fatal self-poisoning are shown in Table 2. The substance most commonly ingested for non-fatal self-poisoning was a pharmaceutical drug (57.1%, *n* = 537). There were significant gender differences in type of substance used (*χ*^2^(1) = 122.7, *p* < .001). Post hoc comparisons of proportions indicated that females were significantly more likely to have ingested a pharmaceutical drug, and significantly less likely than males to have ingested an agrochemical such as an insecticide or pesticide.

**Table 2:** **Substances ingested in non-fatal self-poisoning attempt for the total cohort and for males and females**

**Variable**	**Total sample**	**Gender** ^**1**^		**p value**	**Odds ratio**	**95% CI of OR**
		**Male**	**Female**			
**Type of overdose:**				<.001		
Pharmaceutical drug overdose	55.6% (537)	38.1% (157)	72.0% (380)	na ^2^	na	na
Agrochemical	23.2% (220)	38.1% (157)	11.9% (63)	<.001	0.17	0.12-0.23
Other chemical kept at home	10.6% (101)	12.1% (50)	9.7% (51)	<.001	0.42	0.27-0.65
Plant poison	8.6% (82)	11.7% (48)	6.4% (34)	<.001	0.29	0.18-.047
**Source of substance:**						
Ingested a substance kept at home	59.4% (564)	41.6% (172)	75.5% (392)	<.001	4.33	3.27-5.72
Ingested a substance kept in field/garden	13.7% (130)	21.3% (88)	8.1% (42)	<.001	0.33	0.22-0.48
Bought substance for purpose of ingestion	26.9% (255)	38.3% (158)	18.7% (98)	<.001	0.37	0.28-0.50
**Reason for choice** ^**3**^						
Easy availability	32.3% (307)	28.8% (117)	37.0% (190)	.009	1.45	1.10-1.92
It was cheap	6.4% (61)	6.4% (26)	6.8% (35)	.806	1.07	0.63-1.81
Thought it was not dangerous	11.7% (111)	10.8% (44)	13.0% (67)	.310	1.23	0.82-1.85
Thought it was dangerous	33.1% (314)	37.2% (151)	31.7% (163)	.082	0.78	0.60-1.03
Thought it wouldn’t hurt	13.3% (126)	13.3% (54)	14.0% (72)	.757	1.06	0.73-1.55
Thought it would act quickly	3.6% (34)	5.2% (21)	2.5% (13)	.035	0.48	0.24-0.96

^1^Gender is coded as 0 Male 1 Female.

^2^Not applicable as reference category.

^3^Multiple responses were possible.

Females were significantly more likely than males to ingest a substance that was already available in the home, OR 4.33 (95% CI 3.27 5.72), while males were more likely than females to ingest a substance kept in the field or garden, OR .33 (95% CI .22, .48) or to purchase the substance with the intention of self-poisoning, OR .37 (95% CI .28, .50). Females were also significantly more likely than males to cite ease of availability as a reason for the choice of type of substance ingested, OR 1.45 (95% CI 1.10, 1.92).

### Triggers associated with the non-fatal self-poisoning act

Interpersonal conflict was the most common precipitant or immediate trigger prior to the act of non-fatal self-poisoning – for example, arguments (with spouse/ parent/ other) or problems within a romantic relationship (Table 3). This was reported by both genders. Females were more likely to report a conflict with parents, OR 1.54 (95% CI 1.09, 2.18). Only 3.8% (*n* = 35) of all those who attempted non-fatal self-poisoning reported severe financial difficulties as the precipitant, but of those who did, males were significantly more likely to identify financial difficulties compared to females, OR .07 (95% CI .02, .22).

**Table 3:** **Triggers for non-fatal self-poisoning for the total cohort and males and females**

**Variable**	**Total sample**	**Gender** ^**1**^		**p value**	**Odds ratio**	**95% CI of OR**
		**Male**	**Female**			
Argument with spouse	23.4% (222)	23.4% (95)	24.1% (127)	.791	1.04	0.77-1.41
Argument with parent	17.7% (168)	14.5% (59)	20.7% (109)	.015	1.54	1.09-2.18
Argument with child	1.9% (18)	2.2% (9)	1.7% (9)	.578	0.77	0.30-1.95
Problem with romantic relationship	15.4% (146)	20.0% (81)	12.4% (65)	.002	0.57	0.40-0.81
Severe financial difficulties	3.8% (35)	7.9% (32)	0.6% (3)	<.001	0.07	0.02-0.22

^1^Gender is coded as 0 Male 1 Female.

### Psychiatric morbidity

There was no significant gender difference in depression rates as determined by the PDS and the PHQ-9 (Table 4). More than half of the females (53.7%, *n* = 237) and 49.7% (*n* = 160) of males who had attempted self-poisoning screened positive for depression via the PDS; the PHQ-9 gave comparable results, with 52.0% (*n* = 209) of females and 49.8% (*n* = 152) of males scoring positive for moderate/severe depression. For the subsequent analyses we considered the results for depression as measured by the PDS, since at the time of the conducting the study, the PHQ-9 was not validated for a Sri Lankan population [33]. There was no significant difference in the extent of anxiety between males and females, as measured by the GAD-7, and similarly there was no significant difference in hopelessness scores between the genders, as measured by the BHS.

**Table 4:** **Psychiatric morbidity for the total cohort and for males and females**

Variable*	**Total sample % (n)**	**Gender** ^**1**^		**p value**	**Odds ratio**	**95% CI of OR**
		**Male**	**Female**			
**PDS**						
Depressed	52.0% (397)	49.7% (160)	53.7% (237)	.269	1.18	0.88-1.57
**PHQ-9**						
Moderate/severe depression	51.1% (361)	49.8% (152)	52.0% (209)	.570	1.09	0.81-1.47
**GAD-7**						
Moderate/severe anxiety	39.4% (284)	35.9% (111)	42.1% (173)	.094	1.30	0.96-1.76
**Had history of prior suicide attempts**	13.5% (127)	10.1% (42)	16.2% (85)	.007	1.72	1.16-2.55
**BHS M(SD)**	5.64 (5.36)	5.59 (5.40)	5.67 (5.33)	.822	1.00	0.98-1.03
**AUDIT results:**						
HZD drinking	9.1% (85)	20.7% (85)	0.0% (0)	na^2^	na^2^	na^2^
AUD	3.6% (34)	8.3% (34)	0.0% (0)	na^2^	na^2^	na^2^

^1^Gender is coded as 0 Male 1 Female.

^2^Odds ratio and significance not calculated because no females reported alcohol misuse.

***Notes for abbreviations*** PDS: Peradeniya Depression Scale. Scoring: Total score ≥10/25 is a positive screening for depression.

PHQ-9: Patient Health Questionaire-9. Scoring: 10–19 moderate (includes moderate and moderately severe) depression, 20–27 severe depression.

GAD-7: 7-item anxiety scale. Scoring: 5–9 Mild, 10–14 Moderate, 15–21 severe anxiety.

AUDIT: Alcohol Use Disorders Identification Test. Scoring: ≥7 = Hazardous drinking, ≥16 = Alcohol use disorder.

*Valid data were available for the PDS for 763 participants, for the PHQ-9 for 707 participants, for the GAD-7 for 720 participants, for the AUDIT for 937 participants and for the BHS for 817 participants.

Hazardous drinking and alcohol use disorder was seen among 20.7% (*n* = 85) and 8.3% (*n* = 34) of males respectively, and 24.1% (*n* = 97) of males had ingested alcohol prior to the act of self-poisoning. There were no alcohol use disorders or alcohol ingestion reported among the females. Females (16.2%, *n* = 85) were significantly more likely to have a history of prior suicide attempts compared to males (10.1%, *n* = 42), OR 1.72 (95% CI 1.16, 2.55).

### Stressors in the prior six months and childhood adversity

Stressors occurring within the six months prior to the non-fatal self-poisoning are shown in Table 5. Females were more likely to report a history of interpersonal conflict with parents, OR 1.73 (95% CI 1.20, 2.49), with her father due to his alcohol misuse OR 3.07 (95% CI 1.39, 6.77), and with her spouse because of his alcohol misuse, OR 1.92 (95% CI 1.07, 3.44); they were also significantly more likely to report being a victim of physical abuse in the prior six months, OR 7.02 (95% CI 2.75, 17.90). Males were significantly more likely to report job loss, OR .38 (95% CI .19, .77), financial problems, OR .70 (95% CI .51, .96) and legal problems, OR .04, (95% CI .01, .32).

**Table 5:** **Stressors within the six-months prior to non-fatal self-poisoning**

**Stressors**	**Total sample**	**Gender** ^**1**^		**p value**	**Odds ratio**	**95% CI of OR**
		**Male**	**Female**			
Suffered a serious injury/illness	12.4% (115)	13.3% (54)	11.7% (61)	.481	0.87	0.59-1.29
A close relative had a serious injury/illness	7.1% (66)	5.9% (24)	8.1% (42)	.199	1.41	0.84-2.36
Death of a parent/spouse/child	2.6% (24)	2.5% (10)	2.7% (14)	.816	1.10	0.49-2.51
Death of a friend or close relative	7.5% (69)	6.9% (28)	7.9% (41)	.552	1.16	0.76-1.92
Conflict with parents	16.6% (154)	12.5% (51)	19.8% (103)	.003	1.73	1.20-2.49
Conflict with his/her father because of father’s alcohol use	4.1% (38)	2.0% (8)	5.8% (30)	.005	3.07	1.39-6.77
Conflict with spouse/partner	30.1% (278)	29.6% (120)	30.6% (158)	.741	1.05	0.79-1.39
Conflict with spouse because of alcohol use	6.2% (57)	4.2% (17)	7.8% (40)	.029	1.92	1.07-3.44
Conflict with spouse because of mistrust	9.1% (84)	6.2% (25)	11.5% (59)	.007	1.97	1.21-3.20
Divorce/marital separation	5.0% (46)	4.0% (16)	5.8% (30)	.203	1.50	0.80-2.79
Spouse went overseas for a job	1.6% (15)	1.5% (6)	1.7% (9)	.758	1.18	0.42-3.34
Conflict with a neighbour/friend/relative	5.0% (46)	3.2% (13)	6.4% (33)	.030	2.07	1.07-3.98
Loss of job	3.9% (36)	5.4% (24)	2.3% (12)	.007	0.38	0.19-0.77
Significant financial problems	22.3% (206)	25.8% (105)	19.6% (101)	.024	0.70	0.51-0.96
Court case/legal problems	2.1% (19)	4.4% (18)	0.2% (1)	.002	0.04	0.01-0.32
Seeing bad dreams	10.6% (98)	6.9% (28)	13.6% (70)	.001	2.12	1.34-3.36
Physical abuse within the past 6 months	5.2% (47)	1.3% (5)	8.3% (42)	<.001	7.02	2.75-17.90
Sexual abuse within the past 6 months	1.3% (12)	0.0% (0)	2.4% (12)	na^2^	na^2^	na^2^

^1^Gender is coded as 0 Male 1 Female.

^2^Odds ratio and significance not calculated because no males reported sexual abuse during the past 6 months.

Self-reported childhood adversity is shown in Table 6. There were no significant differences between the genders for most of the items examined under childhood adversity; however, females were significantly more likely to report parental criticisms OR 2.51 (95% CI 1.40, 4.48), harsh punishments OR 2.10 (95% CI 1.04, 4.26), parental separation OR 2.07 (95% CI 1.21, 3.53) and having had a mother working overseas OR 1.70 (95% CI 1.14, 2.55), during their childhood.

**Table 6:** **History of childhood adversity**

**Reported childhood adversity**	**Total sample**	**Gender** ^**1**^		**p value**	**Odds ratio**	**95% CI of OR**
		**Male**	**Female**			
Grew up with constant parental criticism	7.1% (65)	4.0% (16)	9.4% (49)	.002	2.51	1.40-4.48
Was severely punished without reason	4.3% (40)	2.7% (11)	5.6% (29)	.040	2.10	1.04-4.26
Grew up with significant financial problems	31.0% (286)	33.3% (134)	29.2% (152)	.176	0.82	0.62-1.09
Witnessed family members being abused	1.3% 912)	0.5% (2)	1.9% (10)	.057	4.34	0.96-19.69
Has been subjected to physical abuse as a child	4.3% (40)	3.5% (14)	5.0% (26)	.263	1.46	0.75-2.83
Was sexually abused as a child	1.0% (9)	0.5% (2)	1.3% (7)	.212	2.73	0.56-13.21
Had significant problems due to father’s alcohol misuse	17.6% (154)	18.2% (70)	17.2% (84)	.699	0.93	0.66-1.32
Significant parental conflict during childhood	20.9% (182)	20.1% (76)	21.6% (106)	.594	1.09	0.79-1.52
Parents divorced/lived separately	8.1% (71)	5.2% (20)	10.3% (51)	.008	2.07	1.21-3.53
Mother worked abroad during childhood	13.7% (122)	10.3% (40)	16.4% (82)	.010	1.70	1.14-2.55
Mother died before person reached16 years	3.6% (29)	4.5% (16)	2.9% (13)	.217	0.63	0.30-1.32
Father died before person reached 16 years	12.5% (101)	12.1% (43)	12.8% (58)	.789	1.06	0.70-1.62

^1^Gender is coded as 0 Male 1 Female.

### Degree of suicidal intent associated with the act of self-poisoning

Males had significantly higher levels of suicidal intent compared to females OR .92 (95% CI .89, .95) (Table 7). When the total PSIS score was considered by severity category (high -score 11 or over, medium -score 4–10 or low -score below 4), again there was a significant difference between the genders (*χ*^2^(2) = 22.5, *p* < .001); post hoc comparisons of proportions indicated that males were significantly more likely to score high on suicidal intent, whereas females were significantly more likely to have a medium suicide intent.

**Table 7:** **Degree of suicidal intent - Pierce Suicide Intent Scale (PSIS)**

**Variable**	**Total scores**	**Gender** ^**1**^		**p value**	**Odds ratio**	**95% CI of OR**
		**Male**	**Female**			
**Pierce Suicide Intent Scale (PSIS) M(SD)**	10.2 (4.0)	10.9 (4.1)	9.6 (3.83)	<.001	0.92	0.89-0.95
**PSIS subsection scores**						
Circumstances score M(SD)	4.6 (1.9)	4.7 (1.9)	4.53 (1.9)	.107	0.94	0.88-1.01
Self report score M(SD)	4.4 (2.6)	4.8 (2.7)	4.2 (2.6)	<.001	0.91	0.86-0.96
Lethality score M(SD)	1.2 (1.0)	1.5 (1.1)	1.0 (1.0)	<.001	0.63	0.55-.72
**PSIS score by category**				<.001		
Low intent (score 0–3)% (n)	5.4% (51)	5.0% (21)	5.7% (30)	na ^2^	na	na
Medium intent (score 4–10)% (n)	45.2% (429)	37.0% (155)	51.7% (274)	.480	1.24	0.69-2.24
High Intent (score ≥ 11 )% (n)	49.4% (469)	58.0% (243)	42.6% (226)	.151	0.65	0.36-1.17

^1^Gender is coded as 0 Male 1 Female.

^2^Low intent is the reference category.

***Notes for abbreviations:*** PSIS: Pierce Suicide Intent Scale. Total score range: 0–21. Scoring by category: low intent (total score 0–3), medium intent (total score 4–10) and high intent (total score >10).

### Predictors of suicidal intent

The results of multiple regression analysis for males and females are shown in Tables 8 and 9 respectively. Although alcohol use disorders emerged as a significant variable for males, since it was not reported at all for females it could not be included in the final regression models for both genders. The Multiple *R* for the regression models for males and females were significantly different from zero. The adjusted *R*^*2*^ value of .35 for males and .28 for females show that approximately one third of variability of suicidal intent associated with the act of non-fatal self-poisoning was predicted by the models. For both genders, the final block, i.e., psychiatric morbidity (presence of depression and hopelessness scores) contributed most towards the *R*^*2*^_∆_. For males, this contributed 23.9% of the *R*^*2*^_∆_, and for females it contributed 17.2% of the *R*^*2*^_∆_ (*p* < .001).

**Table 8:** **Final stepwise multiple regression model results, examining predictors of suicidal intent associated with non-fatal self-poisoning: males**

**Sets**	**Predictor variables**	**Unstandardized co-efficient**		**Standardized co- efficient**	**t**	**Sig**	**Correlations**		
		**B**	**St error**	**Beta**			**Zero order**	**Partial**	**Part**
Socio demographic variables	Age	.049	.026	.142	1.884	.061	.104	.109	.109
	Marital status	-.475	.612	-.058	-.776	.439	.037	-.045	-.045
Childhood adversity	Parental conflict	1.521	.601	.147	2.529	0.012	.146	.146	.145
	Mother worked overseas	.156	.744	.012	.210	.834	0.017	.012	.012
Stressors during the prior six months	Financial problems	1.389	.556	.151	2.500	.013	.162	.145	.142
	Legal problems	1.458	1.222	.068	1.194	.234	.079	.070	.068
	Physical/sexual abuse	-.420	2.358	-.010	-.178-	.859	.012	-.010	-.010
	Bad dreams	−1.252	.914	-.080	1.369	.172	-.041	-.080	-.078
Prior help seeking/suicidal behaviour	Expressed suicidal ideas	-.383	.677	-.032	-.566	.572	-.012	-.033	-.031
	Saw a doctor in prior month	-.748	.566	-.079	1.322	.187	-.000	-.078	-.073
	Has a past history of suicide attempts	3.611	.810	.254	4.457	.000	.267	.255	.246
Acute triggers	Breakup of romantic relationship	1.500	.605	.154	2.482	.014	.126	.145	.136
Psychiatric morbidity	GAD-7	.025	.057	.033	.438	.662	.401	.026	.020
	PDS	.220	.051	.331	4.281	.000	.487	.247	.200
	BHS	.220	.042	.296	5.227	.000	.469	.297	.244

PDS: Peradeniya Depression Scale, GAD-7: 7 item Anxiety Scale, BHS: Beck Hopelessness Scale.

**Table 9:** **Final stepwise multiple regression model results, examining predictors of suicidal intent associated with non-fatal self-poisoning: females**

**Sets**	**Predictor variables**	**Unstandardized co-efficient**		**Standardized co- efficient**	**t**	**Sig**	**Correlations**		
		**B**	**St error**	**Beta**			**Zero order**	**Partial**	**Part**
Socio demographic variables	Age	.105	.028	.215	3.747	.000	.202	.187	.186
	Marital status	-.191	.445	-.025	-.430	.668	.081	-.023	-.021
Childhood adversity	Parental conflict	1.381	.455	.153	3.037	.003	.154	.153	.149
	Mother worked overseas	.159	.528	.015	.301	.763	.027	.015	.015
Stressors during the prior six months	Financial problems	.831	.511	.084	1.624	.105	.140	.083	.079
	Legal problems	-.653	3.720	-.009	-.176	.861	.021	-.009	-.009
	Physical/sexual abuse	1.821	.671	.136	2.712	.007	.078	.042	.040
	Bad dreams	.443	.538	.041	.823	..411	.153	.137	.132
Prior help seeking/prior suicidal behaviour	Expressed suicidal ideas	.776	.594	.064	1.307	.192	.110	.067	.063
	Saw a doctor in prior month	.500	.394	.062	1.270	.205	.108	.065	.061
	Has a past history of suicide attempts	1.494	.525	.142	2.846	.005	.173	.144	.137
Acute triggers	Breakup of romantic relationship	1.434	.572	.126	2.506	.013	.120	.128	.120
Psychiatric morbidity	GAD-7	.083	.045	.132	1.856	.064	.456	.095	.080
	PDS	.192	.044	.309	4.416	.000	.510	.222	.189
	BHS	.092	.038	.129	2.439	.015	.386	.125	.104

PDS: Peradeniya Depression Scale, GAD-7: 7 item Anxiety Scale, BHS: Beck Hopelessness Scale.

In the final model for males, the degree of suicidal intent was significantly predicted by the following variables: having attempted suicide before (*p* = .006), having expressed suicidal ideas prior to the act (*p* = .025), having visited a doctor in the month prior to the attempt (*p* = .015), seeing bad dreams (*p* = .008), a higher score on the Beck Hopelessness Scale (*p* < .001) and the presence of depression (*p* < .001). For females, in the final model, age (*p* = .015), a higher score on the BHS (*p* = .015), and the presence of depression (*p* < .001), significantly predicted suicidal intent.

## Discussion

Although attempted suicide by non-fatal self-poisoning is common in South Asia and Sri Lanka, little is known about the factors - such as suicidal intent – that are associated with non-fatal self-poisoning in these regions. Our study is the first to explore gender differences in factors associated with higher suicidal intent in non-fatal self-poisoning attempts in Sri Lanka.

Key findings of this study are that a majority of those who present to hospital due to non-fatal self-poisoning are female, and that males and females differ with respect to demographic characteristics, type of substance ingested, alcohol use disorders and suicidal intent. Psychopathology, especially depression, was the most important predictor of suicide intent in both males and females.

### Characteristics of non-fatal self-poisoning

#### Gender distribution

In this study, a greater proportion of females (55.8%) than males presented to hospital due to non-fatal self-poisoning. This is similar to findings from other recent Sri Lankan and Asian studies [13-16,39] and is in accordance with internationally established patterns of higher rates of attempted suicide and self-harm among females [6,17,40]. This finding is unlikely to be a reflection of the distribution of males and females in the population, given that the 2001 population census for Sri Lanka reports a male:female gender ratio of 0.99:1. The higher rate of completed suicides among males may also have influenced this finding [41]. The female predominance in this study is in contrast to several older studies from Sri Lanka, which have reported higher rates of attempted self-poisoning among males [4,5,42,43], and notably in these studies the most commonly ingested substance was pesticides. Similarly, higher rates of attempted self-poisoning among males with regards to pesticide ingestion have been reported in the past by several other regions such as South America, Uganda and India [7,10-12,44].

The female preponderance was more marked in the younger age groups (below 25 year age group male: female ratio = 0.71: 1), whereas there was a male preponderance amongst those aged 35 years and older (35 years and over, male: female ratio = 2.1: 1). This is similar to international patterns [6,45], but our findings are marked in that in the >35 year age group there are more males than females attempting non-fatal self-poisoning.

### Substances used for non-fatal self-poisoning

The most frequent source of self-poisoning was medicinal overdose, in contrast to older studies which report pesticide ingestion as the most common substances used [5], but similar to findings of other more recent Sri Lankan studies [1,14]. This is likely to be a reflection of reduced availability of pesticides, perhaps secondarily to the gradual urbanization of the country. Indeed, both males and females reported that their reason for choice of substance was accessibility. Possible reasons for the higher rate of pesticide ingestion among males could be that in agricultural areas, pesticides are more easily accessible to males who work in the fields, compared to females. The female preponderance of medicinal overdoses has been noted in previous Sri Lankan studies [4,5,39].

While medicinal overdoses generally carry a lower case fatality compared to agrochemical ingestion, the increasing trend of medicinal overdoses, among males and females, is a matter of concern. The question arises as to whether it is timely to consider restriction of sale of over the counter medications (for e.g. paracetamol) in Sri Lanka, similar to the West [46]. However at present Sri Lanka differs from the West in that alternate more toxic substances such as agrochemicals are still fairly widely available, and are still ingested particularly by males, as shown in this study. Furthermore many other types of medications (for e.g., antibiotics, antihypertensives and other similar agents) are also easily available and restrictions regarding the sale of medications in general are not stringent. Therefore a potential risk of restriction of sale of a single medicinal substance (such as paracetamol) is that this may prompt a shift towards self-poisoning with alternate substances with potentially higher lethality, including agrochemical ingestion. Therefore, if restricting availability of medicinal substances, it should be carefully thought out and not randomly implemented. Restriction of the availability of toxic agrochemicals remains a priority given the ongoing agrochemical ingestion, particularly by males [47].

### Psychiatric morbidity

#### Depression

The rates of depression in this study (49.7% of males and 53.7% of females) are much higher than the reported rates for depression in the general population internationally [48,49]. Mood disorders are known important risk factor for suicidal behaviours [50,51] and Hawton et al., in their study of paracetamol overdoses in the UK reported depression to be present in up to 47% of cases [52]. While Asian data is more limited, studies on attempted suicide from India have reported depression rates greater than 30% [2,53-55]. However our findings are in marked contrast to several previous Sri Lankan studies, which have suggested low rates of depression among those who attempt self-poisoning [56,57]. Differences in methodology and the limited number of prior research studies in this area may account for these contrasting findings.

#### Alcohol use disorders

The finding of hazardous drinking or alcohol use disorders in over one-third of males in the current study was in striking contrast to the females, who had no history of alcohol misuse, dependency or ingestion prior to the act. Higher rates of alcohol misuse among males who attempt self-poisoning have been reported previously, both in Sri Lanka and internationally [57,58]. The very marked difference in use of alcohol between males and females in this study is most likely a reflection of alcohol use in the Sri Lankan community [59], where female alcohol use is much less than that of males. Similar patterns have been noted in India as well [54,55]. Reports indicate that the per capita consumption of alcohol in Sri Lanka has increased in recent years, and alcohol misuse and dependency is a major health and social problem among Sri Lankan males [60,61]. Given that alcohol misuse is associated with suicidal behavior [62,63], it is likely to be an important contributory factor towards the non-fatal self-poisoning behavior among males in Sri Lanka. Ongoing alcohol use disorders may be an explanation of why, in the over 35-age group, males in our study outnumbered females with regard to non-fatal self-poisoning. This points to a very important area of intervention for males. Furthermore, Hoek et al. [64] have also described how alcohol misuse among males indirectly contributes towards non-fatal self-poisoning among females, by adding to the likelihood of interpersonal conflict that is often associated with these acts. In keeping with this, although females in our study did not use alcohol, they were significantly more likely than their male counterparts to report interpersonal conflict due to alcohol use by a parent or partner as a stressor during the six months prior to the non-fatal self-poisoning act. Given all of this, the reduction of alcohol use disorders is an important albeit challenging primary preventive measure with regards to prevention of non-fatal self-poisoning. Culturally appropriate interventions to reduce harmful patterns of alcohol within communities maybe a way forwards – for example, via community based education programs [65].

##### Triggers

Common to both genders in this study was the finding that interpersonal conflict was the most commonly identified trigger prior to the act of non-fatal self-poisoning, which is very similar to previous findings from both Sri Lanka and other Asian countries [15,57,66].

### Suicidal intent and predictors of suicidal intent

The males in our study had significantly higher suicide intent than females and it is well documented that suicidal intent at the index episode is associated with later completed suicide [18]. No previous studies from Sri Lanka have compared the degree of gender difference in suicidal intent. Previous international studies comparing intent between genders has reported mixed results, with some studies reporting no difference between the genders [67], while others from the West and South Asia have reported higher intent in males [17,18,54], similar to the findings of our study. However, this is the first time this association has been reported for non-fatal self-poisoning in Sri Lanka.

Despite these gender differences in the characteristics and degree of suicidal intent, the *predictors* of suicidal intent showed more similarities than differences between genders. For both genders, depression and hopelessness were the most important predictors of suicide intent, which is similar to international findings [23,50,68].

Previous studies have suggested that non-fatal self-poisoning in Sri Lanka are mostly impulsive acts, in the context of interpersonal conflict [57]. Based on our findings, we suggest that while most acts of non-fatal self-poisoning do take place in the context of an interpersonal conflict which appear to act as ‘triggers’, a substantial proportion of those attempting poisoning also have associated morbidity such as depression which predicts a greater suicidal intent both for males and females. Similarly in India, depression has been shown to be associated with more high-risk attempts [53,69].

The finding from the current study that the depression is present in 50% of males and over 50% of females and that it is associated with higher suicide intent has important implications given that higher suicidal intent is known to be associated with subsequent completed suicide [18]. In particular it suggests the need for targeted interventions and appropriate management of those who attempt self-poisoning who have psychiatric morbidity. This points to the need for improving mental health literacy in the community and in educating primary care physicians about depression and the role it may play in influencing self-poisoning behaviours.

### Limitations

The findings of this study are based on a cross sectional survey of those admitted to hospital following non-fatal self-poisoning. As a result, those who did not seek hospital care following such an attempt were not included in the analysis. Also, those persons who were not able to speak either Sinhala or English could not be included in the study; this comprised a minority who were only conversant in Tamil, and thus could not be included in the study. A further limitation is that although alcohol misuse was a factor associated in non-fatal self-poisoning in males, alcohol use could not be entered into the multiple regression model, since it was not reported at all among females. However, we have discussed the findings with regard to alcohol use in males separately. Finally certain conditions, such as impulse control disorders and personality disorders were not assessed, and the assessment of psychiatric morbidity was undertaken via screening tools rather than clinician-based diagnostic interviews. However we have attempted to minimize false positives by the use of locally validated screening tools wherever possible.

## Conclusions

The prevention or even minimization of attempted self-poisoning is a complex and challenging task. Understanding and focusing on multiple risk factors, and the interactions between these is likely to be the most useful and cost effective approach to reducing self-poisoning [70,71]. Our findings show that depression and hopelessness predict suicidal intent in both males and females who attempt self-poisoning. For men, prior attempts, and for females, older age also emerge as key factors associated with higher suicidal intent. Since higher suicidal intent is associated with higher risk of completed suicide [18], identification of high intent males and females who have recently attempted self-poisoning, and targeted specific management of such high risk persons maybe an useful intervention at a secondary prevention level.

However primary prevention at a community level remains a challenge. Our data show that although psychiatric morbidity is associated with self-poisoning, about half of those who attempt self-poisoning are *not* depressed; therefore, addressing psychiatric morbidity alone is unlikely to be the answer. This study, as well as previous work, clearly demonstrates interpersonal conflict to be the most common proximal trigger associated with the act of non-fatal self-poisoning, for both males and females. However the females were significantly younger and had a lower suicidal intent, compared to males who were more likely to report an intention to die at the time of the act of self-poisoning. These findings have two implications: first, that interpersonal conflict is a common shared trigger for both genders, but that second, the psychological constructs underlying self-poisoning behaviours may be different for males and females. Marecek et al. [72] in their study of non-fatal self-poisoning among females in Sri Lanka suggested that the act of self-poisoning among girls may represent a form of non-verbal dissent, in situations of interpersonal conflict where norms of obedience and respect constrain overt responses. While beyond the scope of this study, the themes underlying interpersonal conflicts in both genders, and effective ways in which to help young people deal with such interpersonal issues in the community, are areas worth exploring further. There is a need for effective community programs for early development of interpersonal skills in young people, which are both gender and culturally appropriate.

Other population level primary preventive strategies include measures to reduce alcohol misuse and dependency, for e.g., via culturally acceptable community programs to reduce alcohol misuse in males, and increased physician awareness and treatment of primary care depression [65,73]. An integrated, culturally suitable approach is indicated and further research is needed to determine the efficacy of such measures. While our study is based in Sri Lanka, we believe that our findings may have implications for other neighbouring Asian countries which share similar changing patterns non-fatal self-poisoning in recent years.
